# Mortality in Boston in 1843

**Published:** 1844-02

**Authors:** 


*Mortality of Boston in* 1843.—An unusual degree of good health characterized our city the past year. No raging epidemic prevailed, either to alarm the people or sweep off the multitude to a premature grave. The whole number of deaths was 2,197—which was by no means large, in proportion to the population, now believed to be considerably above one hundred thousand. Some of the prominent causes of mortality were as follows, viz: consumption, 249; diseases of the bowels, 65; dropsy of the brain, 85; typhus fever, 72; scarlet fever, 108; infantile diseases, 142; stillborn, 189; measles, 43; inflammation of the lungs, 59; diseases of the heart, 34; convulsions, 50; croup, 52; small-pox, 53.

There is extreme care taken by the city authorities in maintaining an admirable system of street cleanliness; and so long as the same efforts are made to preserve the public health, Boston, we may hope, will continue to be distinguished for its freedom from those pestilential scourges, which occasionally sweep off multitudes in our southern cities.—*Boston Med. and Serg. Journal*, Jan. 1844.

